# Current Insights on Early Life Nutrition and Prevention of Allergy

**DOI:** 10.3389/fped.2020.00448

**Published:** 2020-08-06

**Authors:** Giuliana Ferrante, Maurizio Carta, Claudio Montante, Veronica Notarbartolo, Giovanni Corsello, Mario Giuffrè

**Affiliations:** Dipartimento di Promozione della Salute, Materno-Infantile, Medicina Interna e Specialistica di Eccellenza “G. D'Alessandro,” Università degli Studi di Palermo, Palermo, Italy

**Keywords:** nutrition, allergy, prevention, breastfeeding, microbiota, diet, complementary feeding

## Abstract

The incidence of allergic diseases in childhood appears to have significantly increased over the last decades. Since environmental factors, including diet, have been thought to play a significant role in the development of these diseases, there is great interest in identifying prevention strategies related to early nutritional interventions. Breastfeeding is critical for the immune development of newborns and infants through immune-modulating properties and it impacts the establishment of a healthy gut microbiota. However, the evidence for a protective role of breastfeeding against the development of food allergy in childhood is controversial, and there is little evidence to support the benefits of an antigen avoidance diet during lactation. Although it is not possible to draw a definitive conclusion about the protective role of breast milk against allergic diseases, exclusive breastfeeding is still recommended throughout the first 6 months of life due to associated health benefits. Furthermore, recommendations regarding complementary feeding in infancy have been significantly modified over the last few decades. Several studies have shown that delayed exposure to allergenic foods does not have a role in allergy prevention and recent guidelines recommend against delaying the introduction of complementary foods after 6 months of age, both in high- and low-risk infants. However, trials investigating this dietary approach have reported equivocal results so far. This review summarizes the available high-quality evidence regarding the efficacy of the principal dietary interventions proposed in early life to prevent allergic diseases in children.

## Introduction

Environmental exposures, such as nutritional intake during the critical stages of pregnancy and in the early postnatal period, play a significant role in the development of infant immune system and it has been suspected that may also be involved in the origin of childhood atopic diseases (1).

Many studies have investigated the association between maternal diet and the development of childhood allergic diseases, however, to date, the results have been largely inconclusive and controversial (2). Exclusive breastfeeding is recommended throughout the first 6 months of life, due to associated health benefits, but it is also apparent that breastfeeding should be continued beyond 6 months after the introduction of complementary foods (CF). Duration of breastfeeding rather than exclusivity may be important in prevention of allergic diseases. Nonetheless, while the evidence for a protective role of breastfeeding against the development of food allergy is controversial, the recommendations regarding introduction of complementary foods in infancy have significantly changed over the last decades.

This mini review summarizes the current high-quality evidence regarding the principal early life dietary interventions in preventing allergic diseases in children, particularly in regard to maternal diet during pregnancy, breastfeeding, and introduction of complementary foods.

## Maternal Diet During Pregnancy: Does it Influence the Risk of Allergic Diseases in Offspring?

Maternal diet during pregnancy represents the earliest nutritional exposure to allergens for the fetus. Most studies to date have demonstrated an equivocal correlation between maternal diet during pregnancy and the development of childhood allergic diseases. A Cochrane systematic review did not support allergen avoidance or nutrient supplementation during pregnancy as a means to prevent allergic diseases in offspring (3). However, evidence from mother consumption of peanut and tree nuts suggests that fetal allergen exposure through maternal diet may actually increase tolerance and reduce risk of developing these childhood food allergies (4). Therefore, further studies are needed to elucidate the role of maternal nutrition in the development of food allergies in offspring.

### Fatty Acids

A causal relationship between early maternal consumption of polyunsaturated fatty acids (PUFAs) and childhood allergic diseases is debated (5). The T-helper shift from type 1 to type 2 may derive from a higher n-6: n-3 fatty acids ratio during pregnancy, leading to an increased risk of allergic rhinitis in offspring (6). It is thought that N-3 PUFAs probably limit cytokine cascade (7), decrease n-6 PUFA inflammatory effects, regulate T cell function (cell membrane fluidity, signaling, and gene transcription), and promote long-term effects through epigenetic mechanisms (8). Therefore, daily maternal supplementation of n-3 PUFAs could reduce the risk of food allergies and IgE-associated eczema in infants with a family history of allergy (9). However, findings about the role of n-3 PUFA supplementation in pregnancy for reducing the overall incidence of allergic outcomes in offspring are still insufficient.

### Antioxidants

Antioxidants (i.e., vitamin E, flavonoids, selenium, and copper) intake during pregnancy may be beneficial, as they have immunomodulatory properties and could play a role on fetal lung development and on respiratory health later in life, thus reducing the risk of wheezing (10).

### Vitamin D

The association between vitamin D status of pregnant women and the development of atopic diseases in childhood is not clear. For this reason, prenatal vitamin D supplementation for the prevention of allergic diseases in offspring is not currently recommended. We know 25-hydroxyvitamin D3 levels in cord blood are directly associated with lower mononuclear cell cytokine responses to allergens and reduced risk of eczema in the first 12 months of life (11). In addition, higher vitamin D intake during pregnancy has been associated with lower risk of wheezing and eczema in offspring at 16–24 months of age (12). However, follow-up of the same cohort showed an increased risk of eczema later in life (13). The immunomodulatory mechanism of vitamin D seems to have an influence on Th2 cells differentiation, but its benefits are still unclear and the literature is conflicting (14).

### Foods

A recent prospective birth cohort study showed an association between prenatal maternal intake of certain foods and the risk of allergic diseases in offspring by the age of 3 years (10). Namely, consumption of green vegetables, eggs, and grains were found to play a protective role against respiratory allergic diseases, whereas higher meat intake in the preconception period was positively associated with an increased risk of wheezing, allergic rhinitis, and eczema. In a previous prospective cohort study, a diet rich in vegetables during pregnancy has been associated with reduced risk of childhood asthma in offspring (15). A systematic review and meta-analysis concluded that maternal fish consumption during pregnancy did not reduce the risk of allergic outcome in offspring (16).

## Breastfeeding: Does Human Milk Play a Protective Role?

Current guidelines recommend human milk (HM) as the “gold standard” for infant nutrition (17). Breastfeeding prolongs the interaction with the mother's immune system and may influence oral tolerance and the risk of allergies in childhood. Pivotal studies clearly confirm the relevant role of breastfeeding for short-term infant health (e.g., growth, immune function, protection against infections) as well as for potential long-term advantages (e.g., neurocognitive development, prevention of malignancies, and non-communicable diseases) (18). Nevertheless, the evidence for the role of HM for the prevention of allergic diseases remains limited and controversial. This is due to variable definitions of allergic outcomes and to the lack of randomized controlled trials with detailed information about the maternal diet in breastfeeding. While many studies emphasize a protective effect, others even suspect HM may promote allergies (19). The balance between oral tolerance and skin sensitization may affect food allergy risk among infants with eczema: additional evidence suggests that colostrum has a prophylactic role in maintaining oral tolerance, but in case of severe cutaneous barrier dysfunction the protective effect of prolonged breastfeeding is lost (20). Early oral exposure to aeroallergens through HM intake could increase the risk of sensitization in offspring (21). On the other hand, exclusive breastfeeding seems to reduce the incidence of eczema in the first 2 years of life and the risk of asthma in the first 5 years (22, 23), or even 10 years with weaker evidence (24).

Several reasonable explanations account for HM protective effects on allergy susceptibility in children: stimulation of immune development (direct interactions with infant immune cells), epigenetic actions (DNA methylation, and non-coding RNAs), modulation of gut microbiota.

Breast milk is a living tissue and various bioactive factors appear to be involved. For instance, TGF-β is a regulatory cytokine crucial for long-lasting Treg-mediated food tolerance (25). Maternal antigen immune complexes (IgG-IC) in breast milk may interact with the neonatal crystallizable fragment receptor (FcRn) on infant dendritic cells (26), favoring oral tolerance. In addition, breast milk contains up to 10^5^ bacterial cells/ml: HM microbiota plays a role in bacterial colonization of the infant intestinal tract that could contribute to modulate allergy susceptibility early and later in life. Finally, human milk oligosaccharides (HMOs) can play a key role in neonatal mucosal and systemic immunity. HMOs represent an antimicrobial barrier acting as soluble decoy receptors that block adhesion of various pathogens, promote intestinal development, and stimulate immunomodulation acting as signal molecules for the host cells. Some HMOs have been proven to play a crucial role in shaping gut microbiota and regulating early life immune development, but the precise mechanism is currently unknown. Furthermore, specific HMOs profiles have been associated with lower risk of cow's milk allergy (27).

Different formulas are available for newborns and infants who cannot be breastfed. Among them, hydrolyzed formulas have been specifically proposed for infants at risk of allergies; however, their role in allergy prevention is still under debate (28).

## Complementary Feeding: Does Timing of Food Introduction Influence the Risk of Allergies?

In past years, it has been traditionally recommended to delay the introduction of foods recognized as potentially allergenic, based on the theory that the gut structural and functional immaturity and increased permeability determines an increased risk of allergic sensitization (29). Nevertheless, early exposure to these allergens may be critical to achieve food tolerance, which is an antigen-driven process as suggested by animal models (30). According to the so-called “dual-allergen exposure hypothesis,” oral administration of food allergens favors the establishment of tolerance via the expansion of Th1 and Treg populations, whereas exposure to food allergens through skin barrier disruptions favors sensitization via Th2 switch and cytokines production (29).

Current evidence suggests that oral allergen exposure may start at 4 months onwards, although proper timing of this “critical window” is still not clear (31).

In 2016, the Enquiring About Tolerance trial showed that, in a general population of exclusively breastfed infants, the early (i.e., between 3 and 6 months of age) introduction of potentially allergenic foods (cow's milk, cooked egg, peanut, fish, wheat, and sesame) was effective for prevention of food allergy (32). In particular, while the incidence of food allergy at 3 years of age was not significantly different between the early introduction group compared to the control group in the intention-to-treat analysis, the per-protocol analysis showed a significant reduction of overall incidence of food allergy, peanut allergy, and egg allergy in the early introduction group. However, the high rate of non-adherence in the early introduction group (68.1%) limits the reliability of the study results. Results from the Learning Early About Peanut Allergy trial suggested that peanut should be introduced between 4 and 11 months in infants at high risk for allergy (33). According to a recent systematic review, this practice may reduce the risk of peanut allergy, with the strongest evidence supporting a benefit to high risk infants (i.e., with severe atopic dermatitis or egg allergy) but it is also applicable to infants at lower risk (34). In addition to these findings, there is limited high quality evidence suggesting the lack of a relationship between consumption of peanut during weaning and the risk of atopic dermatitis/eczema and asthma, while not enough evidence supports the causal relationship between consuming peanut and developing allergic rhinitis at 2, 5, or 6 years of age (34).

Many studies addressing the effect of egg introduction on the risk of developing allergy, have provided conflicting results. This is likely due to unaccounted variables from different study populations, as well as variations in dose and form of the eggs used (raw or cooked) (35–39). However, a recent systematic review provided moderate evidence that introducing egg at 4–6 months of age may reduce the risk of developing egg allergy (34). In the same study, limited evidence showed the lack of association between the timing of egg introduction and the development of atopic dermatitis/eczema and asthma, while not enough evidence supported a relationship with risk of allergic rhinitis in the first 5 years of life (34).

Previous observational studies have also investigated the effect of the timing of fish introduction on the risk of allergic diseases (40, 41). A recent systematic review provided limited evidence to suggest that the introduction of fish between 3 and 8 months of life may lower the risk of atopic dermatitis/eczema (34). However, at present there is not enough evidence of a link between fish consumption and food allergy, asthma, or allergic rhinitis (42).

With regard to cow-milk, while previous observational studies reported conflicting results on the risk of cow's milk allergy (43, 44), more recently a randomized clinical trial demonstrated that the risk of sensitization to cow's milk as well as the risk of immediate food allergy were decreased by avoiding supplementation with cow's milk formula for at least the first 3 days of life (45). In addition, a recent systematic review provided limited high quality evidence suggesting the lack of a relationship between age of introduction and risk of food allergy and atopic dermatitis/eczema. However, there was not enough evidence to support an association between cow's milk formula supplementation and the development of asthma and allergic rhinitis (34).

Overall, these findings suggest that there is not a clear optimal timing of introduction of potential allergenic foods for preventing allergic diseases, either for infants in the general population or for high-risk infants (Table 1). The latest recommendations issued by the World Health Organization (46), the American Academy of Pediatrics (22), the European Academy of Allergy and Clinical Immunology (47), the European Society for Paediatric Gastroenterology Hepatology and Nutrition (30), the European Food Safety Authority (48), and the British Society for Allergy, and Clinical Immunology (49) have all emphasized the lack of scientific data to support the introduction of the commonly acknowledged allergenic foods before 4 months of age, but also the absence of evidence to justify a delayed introduction of CF after 6 months of age to prevent allergy, both in high and low-risk infants (Supplementary Table 1).

**Table 1:** Timing of introduction of CF and risk of allergic diseases in childhood.

**Type of CF**	**Allergic disease**			
	**Food allergy**	**Atopic dermatitis/eczema**	**Asthma**	**Allergic rhinitis**
Peanut	Introduction between 4 and 11 months may decrease the risk of peanut allergy in infants at high and low risk (34^*^, 35^#^)	No relationship between consumption during weaning and the risk of atopic dermatitis/eczema (35^#^)	No relationship between consumption during weaning and the risk of asthma (35^#^)	No relationship between consumption during weaning and developing allergic rhinitis (35^#^)
Egg	Introduction between 4 to 8 months does not prevent egg allergy in high risk-infants (37^*^, 39^*^) Introduction between 4 to 6 months does not prevent egg allergy in low and high risk-infants (36^*^, 38^*^) Introduction between 3 to 9 months is effective in preventing egg allergy in high risk-infants (40^*^) Introduction between 4 and 6 months may decrease the risk of allergy to egg (35 ^#^)	No relationship between consumption during weaning and the risk of atopic dermatitis/eczema (35^#^)	No relationship between consumption during weaning and the risk of asthma (35^#^)	No relationship between consumption during weaning and developing allergic rhinitis (35^#^)
Fish	Fish consumption during weaning is not associated with increased risk of food allergy (35^#^)	Introduction between 3 and 8 months of age may decrease the risk of atopic dermatitis/eczema (35^#^, 42^§^)	No relationship between consumption during weaning and the risk of asthma (35^#^, 43^#^)	Introduction before 9 months of age may reduce the risk of allergic rhinitis (41^§^) No relationship between consumption during weaning and the risk of allergic rhinitis (35 ^#^)
Cow-milk products	Introduction within the first few days of life is associated with an increased risk of developing cow's milk allergy (44^^§^^) Introduction within the first 2 weeks of life reduces the risk of cow's milk allergy, whereas introduction between 4 to 6 months increased this risk (45^§^) Avoiding supplementation with cow's milk formula for at least 3 days of life decreases the risk of cow's milk allergy (46^*^) No relationship between age of introduction and risk of food allergy (35 ^#^)	No relationship between age of introduction and risk of atopic dermatitis/eczema (35^#^)	No relationship between age of introduction and risk of asthma (35^#^)	No relationship between age of introduction and risk of allergic rhinitis (35^#^)

§ *observational study*.

* *randomized control study*.

# *systematic review*.

## Diet-Induced Changes in gut Microbiota: Do They Influence Allergy Risk?

### Pregnancy

Evidence suggests that early life gut microbial dysbiosis precedes atopy development and that the gut microbiota of allergic children may show a depleted diversity (50, 51). In particular, a reduction of certain bacteria (e.g., *Bifidobacterium, Akkermansia*, and *Faecalibacterium*) could drive CD4+ cell dysfunction, thereby increasing the risk of atopic diseases (52). It has been recently suggested that diet during pregnancy may indirectly affect tolerance acting on the microbiota diversity. In particular, fiber consumption appears to lead to changes in the maternal gut microbiota metabolism by increasing the production of short-chain fatty acids (SCFAs), which represent the first metabolites of gut commensal microbiota. Therefore, SCFAs that cross the placenta, influence gene transcription in fetal lung and enhance oral tolerance to allergens by promoting epithelial integrity, T regulatory (Treg) cell differentiation, and IgA release from plasma cells, with long-lasting effects on health (53). Furthermore, it seems that fetal Treg cells may be informed by substantial numbers of maternal cells crossing the placenta and inducing antigen-specific tolerance. *In utero* transfer of microbial antigens during fetal development would enable a balanced immune response of the newborn to the rapidly developing microbiota *post-partum* (54).

### Early Life

In recent years there has been increased scientific interest and studies into the role of CF introduction to the infant diet and the risk of allergy. Investigations have sought to determine if there is a cause–effect relationship between diet-induced changes of infant's microbiota and the development of allergic diseases.

Indeed, during weaning, a dramatic change of intestinal microbiota occurs, which is reflected in the transitioning nature of stool composition (55). Thus, it has been suggested that the resident microbiota of the gastrointestinal tract, interacting with solid foods, may be able to modulate the immune system development in early life (56).

In breastfed infants, a rapid rise in the number of *Enterobacteria* and *Enterococci* has been demonstrated (57), along with an increased amount of *Bifidobacterium* and *Lactobacillus* spp. (58). Conversely, in formula-fed infants, a greater abundance of *Bacteroides* and *Clostridium* spp. has been observed, this difference could have implications for the subsequent development of atopic diseases (57, 58). The introduction of CF drives the gut microbiota composition favoring bacteria within the *Bacteroidetes* and *Firmicutes phyla* (58). Most of the reported changes in gut microbiota appear to occur after weaning, from age 9–18 months until the 3rd year of life, when the microbiota stabilizes resembling the adult one (59). Therefore, the window of opportunity for modulating the intestinal microbiota composition extends up to the first 2 years of life, conversely any dysbiosis in this period could determine an abnormal immune system activation, possibly leading to the development of pathological conditions such as allergy (56).

It has been hypothesized that a more diverse diet leads to a more diverse gut microbiota, which may improve the gut wall integrity and the immune system regulation, supporting the expansion of Treg cells (60), and suppressing IgE levels (61). Indeed, a lower microbiota diversity with an increased number of *Firmicutes* vs. of *Bacteroidetes*, has been observed in children with food sensitization (62). An increased microbial diversity along with the relative abundance of certain bacteria, such as *Lactobacillus* spp. (63), has been correlated with a lower risk of IgE-associated allergic diseases, namely atopic dermatitis and wheezing, through a decreasing Th2-mediated response (64). No significant association between the diversity of complementary foods and allergic rhinitis has been found so far (65).

According to these findings, early infancy does appear as a window of opportunity during which diet intervention may shape the risk of allergic diseases by modulating the composition of the gut microbiota (60). In this context, it has been demonstrated in a murine model that dietary fiber intake leads to marked suppression of the induction of airway allergic disease, by enhancing Treg cell number and function (66). A possible explanation is that SCFAs, which would seem to reduce airway inflammation even in human models, are produced by the microbiota through the metabolism of dietary fibers (65).

A protective effect against the risk of allergic diseases might be also derived from the supplementation with prebiotics which can indirectly promote the production of anti-inflammatory cytokines by increasing the number of *Lactobacillus* and *Bifidobacterium* spp. (67). Finally, it has been suggested that adequate levels of vitamin D during the 1st year of life may lower the risk of developing food allergies, by modulating the gut microbiota composition (68), with increased *Lachnospiraceae* and reduced *Lactococcus* spp. (69).

Overall, these studies have contributed to deepening our knowledge about gut microbiota diversity and species-specific changes. Unfortunately, however, how to manipulate the intestinal microbiota for the prevention of allergic diseases is still a matter of debate.

## Conclusions

**Figure 1:**
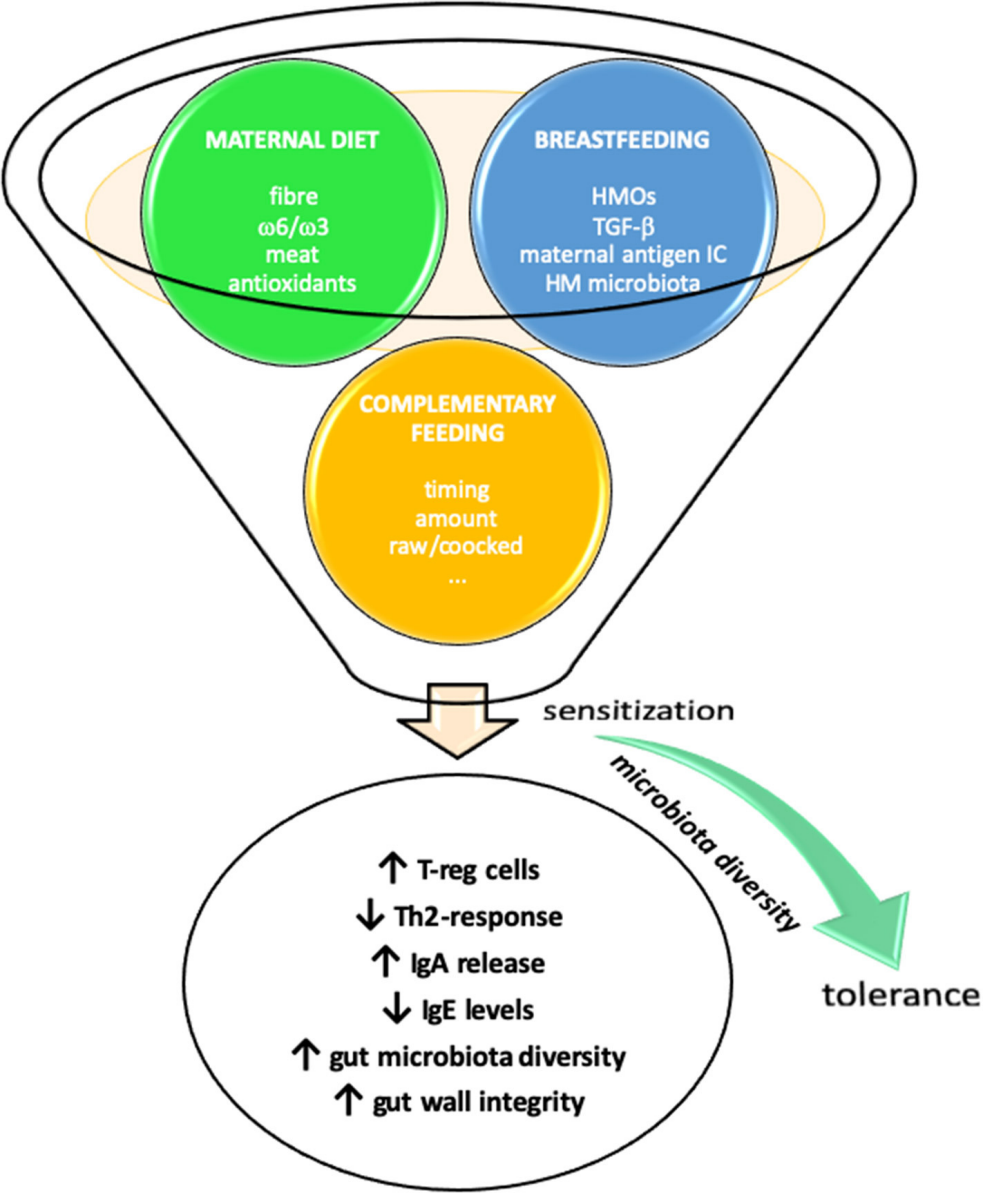
Early life nutrition and prevention of allergic diseases. Maternal diet during pregnancy and lactation and introduction of complementary foods may influence susceptibility to allergic diseases through several diverse mechanisms.

The link between early dietary factors and the development of allergy later in life is still not clear. Many dietary factors, from prenatal life through infancy, have been proposed to influence the susceptibility to allergic diseases, by modulating the gut microbiota composition and promoting tolerance to allergens. None of these, other than early introduction of allergens, has been proven effective (Figure 1).

Current high-quality evidence for the efficacy of the principal dietary interventions demonstrates that maternal avoidance of allergenic foods during pregnancy and lactation is not effective in preventing allergic diseases. Some evidence suggests that a maternal diet rich in fibers, antioxidants, and n-3 PUFAs might promote a protective immunomodulatory role, also mediated by changes in microbiota. However, further studies are needed to elucidate the role of maternal nutrition in the development of food allergy in offspring.

Evidence for an overall protective role of breastfeeding against allergic diseases is still controversial, even though exclusive breastfeeding is recommended as the “gold standard” for infant nutrition during the first 6 months of life.

With regard to complementary feeding, there is no clear evidence to support a specific timing to the introduction of potentially allergenic foods for preventing allergic diseases, either for infants in the general population or for high-risk infants. Nonetheless, complementary feeding should not be delayed after 6 months of age, nor should breastfeeding be discontinued.

The diversity of the intestinal microbiota in early life is likely associated with a reduced risk of allergies. Indeed, it has been suggested that poor gut microbial diversity may increase the risk of developing allergic diseases. Thus, early infancy does appear as a window of opportunity during which dietary intervention may influence the risk of allergic diseases.

To better identify efficacious dietary strategies for primary prevention of allergies in childhood, future research should implement longitudinal interventional studies in cohorts of pregnant women and their offspring, as well as randomized controlled trials to clarify the potential role of complementary foods and their optimal timing of introduction.

## Author Contributions

GF, MC, and MG: conceptualization. GF, MC, CM, VN, and MG: writing original draft. GC and MG: review and editing. All the authors read and approved the final version of the manuscript.

## Conflict of Interest

The authors declare that the research was conducted in the absence of any commercial or financial relationships that could be construed as a potential conflict of interest.
