# Supplementary material for: Current Insights on Early Life Nutrition and Prevention of Allergy
Source: Front Pediatr. 2020 Aug 6;8:448. doi: 10.3389/fped.2020.00448 (PMC7424002; doi:10.3389/fped.2020.00448)
Supplement: Supplementary file 1 [file Table_1.docx]

**Table S1.** Summary of guidelines for timing of complementary food introduction.

| **Organization** | **Guidance** |
| --- | --- |
| World Health Organization (WHO) (47) | All infants should receive complementary foods in addition to human milk starting from 6 months onwards. |
| American Academy of Pediatrics (AAP) (23) | Complementary foods should not be introduced before 4 to 6 months of age. Delaying the introduction of allergenic foods beyond 4-6 months does not prevent the development of atopic diseases.  **Peanuts:** early introduction reduces the risk for peanut allergies. Testing before introduction in high risk infants is recommended.  **Egg:** data are less clear for timing of introduction. |
| European Academy of Allergy and Clinical Immunology (EAACI) (48) | Introduction of complementary foods after the age of 4 months for all infants, irrespective of atopic heredity. |
| European Society for Pediatric Gastroenterology, Hepatology, and Nutrition (ESPGHAN) (31) | Introducing complementary foods before 3-4 months may increase the risk of allergy. However, delaying the introduction of allergenic foods beyond 4-6 months does not prevent the development of atopic diseases, either for infants in the general population or for those with a family history of atopy.  **Peanuts:** in infants at high risk of peanut allergy (i.e. those with severe eczema, egg allergy, or both) should receive peanuts between 4 to 11 months, after evaluation by an appropriately trained professional. |
| European Food Safety Authority (EFSA) (49) | Allergenic foods should be introduced along with other complementary foods between the age of 4 to 6 months.  **Peanuts:** introduction during the ﬁrst year of life reduces the risk of peanut allergy.  **Egg:** cooked egg can be introduced when other complementary foods are introduced. |
| British Society for Allergy and Clinical Immunology (BSACI) (50) | All infants should receive complementary foods at around 6 months of age alongside breastfeeding, but not before 4 months.  **Peanuts and egg:** delaying the introduction beyond 6 to 12 months may increase the risk of allergy. Infants at high risk (i.e. with eczema and/or food allergy) may beneﬁt from the introduction of cooked egg (and then peanuts), alongside other solids from the age of 4 months. |
